# Supplementary material for: Gender Differences in the Impact of New-Onset Atrial Fibrillation on Long-Term Risk of Ischemic Stroke after Acute Myocardial Infarction
Source: J Clin Med. 2021 Nov 1;10(21):5141. doi: 10.3390/jcm10215141 (PMC8584956; doi:10.3390/jcm10215141)
Supplement: Supplementary file 1 [file jcm-10-05141-s001.zip › jcm-1437043-supplementary.pdf]

Table S1. Independent predictors of ischemic stroke after AMI.

Variables	Univariate Analysis		Multivariate Analysis		
	HR	95% CI	HR	95% CI	*p*
**Overall population**					
Non-AF	1 (reference)		1 (reference)		NA
NOAF	1.983	1.175–3.346	1.874	1.086–3.235	0.024
Previous AF	5.969	3.335–10.685	4.001	2.034–7.869	<0.001
Age	1.041	1.030–1.053	1.038	1.024–1.052	<0.001
Women	1.458	1.119–1.900			
Body mass index (kg/m^2)	0.932	0.893–0.971	0.957	0.916–1.000	0.051
Diabetes mellitus	1.489	1.150–1.929	1.421	1.077–1.876	0.013
Hypertension	1.587	1.228–2.052	1.293	0.966–1.732	0.084
Dyslipidemia	0.918	0.638–1.320			
Chronic kidney disease	1.490	0.554–4.008			
Current smoker	0.834	0.645–1.079	1.416	1.047–1.915	0.024
Previous HF	2.522	1.039–6.121			
Previous MI	1.569	0.915–2.690			
Previous stroke	2.553	1.777–3.668	2.072	1.403–3.059	<0.001
Killip class ≥ 2	1.516	1.134–2.028			
STEMI (vs. NSTEMI)	0.924	0.718–1.189			
Extent of CAD					
1 vessel disease	1 (reference)		1 (reference)		NA
2 vessel disease	1.171	0.876–1.566			
3 vessel disease	1.279	0.930–1.759			
DAPT at discharge	1.023	0.525–1.994			
Anticoagulant at discharge	1.773	0.911–3.451			
**Men**					
Non-AF	1 (reference)		1 (reference)		NA
NOAF	1.428	0.669–3.048	1.386	0.606–3.167	0.439
Previous AF	5.246	2.316–11.880	4.135	1.791–9.546	0.001
Age	1.039	1.026–1.053	1.036	1.049–1.053	<0.001
Body mass index (kg/m^2)	0.912	0.864–0.962	0.940	0.886–0.997	0.041
Diabetes mellitus	1.710	1.243–2.353	1.591	1.128–2.243	0.008
Hypertension	1.563	1.147–2.130	1.329	0.935–1.889	0.113
Dyslipidemia	0.883	0.558–1.399			
Chronic kidney disease	1.823	0.581–5.725			
Current smoker	0.953	0.700–1.298	1.528	1.073–2.177	0.019
Previous HF	2.095	0.519–8.457			
Previous MI	1.561	0.823–2.961			
Previous stroke	2.940	1.891–4.572	2.181	1.337–3.558	0.002
Killip class ≥ 2	1.190	0.801–1.768			
STEMI (vs. NSTEMI)	0.983	0.719–1.344			
Extent of CAD					
1 vessel disease	1 (reference)		1 (reference)		NA
2 vessel disease	1.080	0.748–1.559	0.820	0.549–1.224	0.331
3 vessel disease	1.605	1.101–2.339	1.309	0.878–1.951	0.187
DAPT at discharge	0.695	0.340–1.418	0.661	0.320–1.363	0.262
Anticoagulant at discharge	1.634	0.671–3.982			
**Women**					
Non-AF	1 (reference)		1 (reference)		NA
NOAF	2.845	1.368–5.916	2.543	1.209–5.352	0.014

Previous AF	6.464	2.802–14.911	3.719	1.156–11.962	0.028
Age	1.049	1.024–1.074	1.035	1.008–1.062	0.009
Body mass index (kg/m^2)	0.978	0.917–1.043			
Diabetes mellitus	1.052	0.676–1.637			
Hypertension	1.391	0.860–2.249			
Dyslipidemia	0.962	0.532–1.741			
Chronic kidney disease	0.941	0.131–6.781			
Current smoker	0.949	0.458–1.967			
Previous HF	2.512	0.791–7.974			
Previous MI	1.764	0.646–4.820			
Previous stroke	1.840	0.975–3.471	1.897	0.996–3.612	0.051
Menopause	1.605	0.950–2.711			
Killip class ≥ 2	1.953	1.245–3.063	1.736	1.081–2.788	0.023
STEMI (vs. NSTEMI)	0.885	0.574–1.363			
Extent of CAD					
1 vessel disease	1 (reference)		1 (reference)		NA
2 vessel disease	1.293	0.804–2.080			
3 vessel disease	0.716	0.390–1.314			
DAPT at discharge	3.764	0.523–27.069			
Anticoagulant at discharge	1.813	0.664–4.952			

Table S2. Characteristics according to onset time of AF in the overall population (n = 10,137).

Variables	Non-AF (n = 9,637)	NOAF (n = 370)	Previous AF (n = 130)	p Value
Age (years)	62.9 ± 12.7	68.4 ± 12.1 *	71.6 ± 9.2 †,‡	<0.001
Women (%)	2642 (27.4)	118 (31.9)	50 (38.5)	0.004
Body mass index (kg/m2)	24.2 ± 3.3	24.0 ± 3.4	23.8 ± 3.9	0.167
Diabetes mellitus (%)	3004 (31.2)	109 (29.5)	55 (42.3)	0.019
Hypertension (%)	4945 (51.3)	213 (57.6)	92 (70.8)	<0.001
Dyslipidemia (%)	1575 (16.3)	43 (11.6)	17 (13.1)	0.034
Chronic kidney disease (%)	179 (1.9)	11 (3.0)	8 (6.2)	0.003
Current smoker (%)	3986 (41.4)	129 (34.9)	21 (16.2)	<0.001
Family History of CAD (%)	294 (3.1)	5 (1.4)	1 (0.8)	0.055
Previous HF (%)	97 (1.0)	14 (3.8)	23 (17.7)	<0.001
Previous MI (%)	380 (3.9)	28 (7.6)	8 (6.2)	0.001
Previous PAD (%)	48 (0.5)	5 (1.4)	2 (1.5)	0.025
Previous stroke (%)	649 (6.7)	37 (10.0)	21 (16.2)	<0.001
SBP at admission (mmHg)	130 ± 26	122 ± 29*	127 ± 29	<0.001
DBP at admission (mmHg)	79 ± 16	75 ± 18*	78 ± 18	<0.001
HR at admission (bpm)	78 ± 18	82 ± 24*	84 ± 25†	<0.001
Killip class ≥ 2 (%)	2122 (22.0)	177 (47.8)	52 (40.3)	<0.001
LV ejection fraction (%)	53.6 ± 11.0	49.8 ± 12.6 *	49.5 ± 11.5†	<0.001
LA AP diameter (mm)	36.7 ± 5.9	40.2 ± 8.3 *	46.6 ± 8.4†,‡	<0.001
$eGFR_{MDRD}$ ($mL/m^2/1.73m^2$)	64.6 ± 23.8	56.2 ± 23.7 *	50.0 ± 22.4†,‡	<0.001
HbA1c (%)	6.6 ± 1.6	6.4 ± 1.3	6.7 ± 1.6	0.095
Total cholesterol (mg/dL)	178.8 ± 43.3	165.8 ± 42.1 *	156.8 ± 43.6†	<0.001
Triglyceride (mg/dL)	125.8 ± 93.5	105.1 ± 67.2 *	92.2 ± 53.7†	<0.001
HDL cholesterol (mg/dL)	40.9 ± 10.9	41.1 ± 11.2	41.5 ± 11.2	0.844
LDL cholesterol (mg/dL)	114.2 ± 37.9	103.2 ± 37.3 *	100.0 ± 36.0 †	<0.001
Peak CK (U/L)	444 (150,1455)	473 (141,1366)	316 (132,784) †,‡	0.003
Peak CK-MB (μg/mL)	48.2 (14.0,146.3)	41.7 (15.6,125.6)	29.1 (7.7,110.3) ‡	0.019
Peak TnI (ng/mL)	22.7 (4.4,52.5)	20.8 (5.6,50.0)	19.4 (2.1,53.9)	0.188
STEMI (%)	5180 (53.8)	195 (52.7)	55 (42.3)	0.032
Extent of CAD (%)				
One vessel disease	4412 (45.8)	191 (51.6)	55 (42.3)	0.061
Two vessel disease	3144 (32.6)	122 (33.0)	44 (33.8)	0.949
Three vessel disease	2081 (21.6)	57 (15.4)	31 (23.8)	0.014
CABG (%)	19 (0.2)	1 (0.3)	0 (0.0)	0.637
CHA_2DS_2-VASc score	2.4 ± 2.0	3.4 ± 2.1 *	4.1 ± 2.1 †,‡	<0.001
Medications at discharge (%)				
Aspirin (%)	9479 (98.4)	362 (97.8)	123 (94.6)	0.010
P_2Y_{12} inhibitor (%)	9207 (95.5)	362 (97.8)	121 (93.1)	0.039
DAPT (%)	9147 (94.9)	355 (96.2)	118 (90.6)	0.046
Anticoagulant (%)	156 (1.6)	61 (16.6)	40 (30.8)	<0.001
Warfarin	144 (1.5)	60 (16.3)	39 (30.0)	<0.001
NOAC	12 (0.1)	1 (0.3)	1 (0.8)	0.084
DAPT plus anticoagulant (%)	141 (1.5)	58 (15.7)	37 (28.5)	<0.001
Statin (%)	9256 (96.0)	363 (98.1)	122 (94.4)	0.075
Beta-blocker (%)	8400 (87.2)	312 (84.4)	107 (82.8)	0.123
ACE inhibitor or ARB (%)	8798 (91.3)	350 (94.7)	117 (90.5)	0.101

* $p < 0.05$ between Non-AF vs. NOAF; † $p < 0.05$ between Non-AF vs. Previous AF; ‡ $p < 0.05$ between NOAF vs. Previous AF.

Table S3. Characteristics according to onset time of AF in men (n = 7327).

Variables	Non-AF (n = 6,995)	NOAF (n = 252)	Previous AF (n = 80)	p Value
Age (years)	59.9 ± 12.2	65.7 ± 12.4 *	69.1 ± 9.8 †,‡	<0.001
Body mass index (kg/m²)	24.4 ± 3.1	24.3 ± 3.2	23.3 ± 2.7 †	0.006
Diabetes mellitus (%)	1978 (28.3)	70 (27.8)	31 (38.8)	0.116
Hypertension (%)	3,188 (45.6)	124 (49.2)	55 (68.8)	<0.001
Dyslipidemia (%)	1121 (16.0)	23 (9.1)	12 (15.0)	0.013
Chronic kidney disease (%)	120 (1.7)	9 (3.6)	3 (3.8)	0.039
Current smoker (%)	3726 (53.3)	118 (46.8)	20 (25.0)	<0.001
Family History of CAD (%)	246 (3.5)	5 (2.0)	0 (0.0)	0.102
Previous HF (%)	40 (0.6)	9 (3.6)	16 (20.0)	<0.001
Previous MI (%)	292 (4.2)	21 (8.3)	6 (7.5)	0.004
Previous PAD (%)	38 (0.5)	3 (1.2)	2 (2.5)	0.037
Previous stroke (%)	427 (6.1)	23 (9.1)	14 (17.5)	<0.001
SBP at admission (mmHg)	130 ± 26	123 ± 29 *	122 ± 27†	<0.001
DBP at admission (mmHg)	80 ± 16	76 ± 18 *	74 ± 18†	<0.001
HR at admission (bpm)	78 ± 18	81 ± 22 *	82 ± 24	0.003
Killip class ≥ 2 (%)	1382 (19.8)	115 (46.0)	34 (42.7)	<0.001
LV ejection fraction (%)	53.9 ± 10.8	49.0 ± 12.5 *	49.2 ± 11.0†	<0.001
LA AP diameter (mm)	36.7 ± 5.8	40.7 ± 8.3 *	46.2 ± 8.8†,‡	<0.001
$eGFR_{MDRD}$ (mL/m²/1.73m²)	62.7 ± 21.9	51.1 ± 21.2 *	48.2 ± 19.9†	<0.001
HbA1c (%)	6.6 ± 1.6	6.3 ± 1.3	6.6 ± 1.8	0.086
Total cholesterol (mg/dL)	177.6 ± 41.9	163.8 ± 39.6 *	152.8 ± 40.4	<0.001
Triglyceride (mg/dL)	129.5 ± 100.4	109.9 ± 73.0 *	84.5 ± 45.5†	<0.001
HDL cholesterol (mg/dL)	40.1 ± 10.4	40.6 ± 11.0	40.7 ± 11.9	0.693
LDL cholesterol (mg/dL)	113.5 ± 36.8	100.7 ± 33.9 *	97.6 ± 33.0†	<0.001
Peak CK (U/L)	491 (160,1630)	529 (151,1392)	426 (153,886)	0.152
Peak CK-MB (μg/mL)	55.4 (15.2,156.4)	44.8 (17.9,146.0)	51.0 (12.7,169.7)	0.533
Peak TnI (ng/mL)	25.0 (4.9,55.6)	22.8 (8.1,50.0)	31.7 (7.2,72.3)	0.539
STEMI (%)	3913 (55.9)	143 (56.7)	35 (43.8)	0.088
Extent of CAD (%)				
One vessel disease	3312 (47.3)	136 (54.0)	42 (52.5)	0.080
Two vessel disease	2281 (32.6)	79 (31.3)	22 (27.5)	0.577
Three vessel disease	1402 (20.0)	37 (14.7)	16 (20.0)	0.111
CABG (%)	13 (0.2)	1 (0.4)	0 (0.0)	0.478
CHA_2DS_2-VASc score	1.8 ± 1.6	2.7 ± 1.9 *	3.5 ± 2.0 †,‡	<0.001
Medications at discharge (%)				
Aspirin (%)	6883 (98.4)	249 (98.8)	77 (96.3)	0.240
P_2Y_{12} inhibitor (%)	6685 (95.6)	246 (97.6)	73 (91.3)	0.043
DAPT (%)	6650 (95.1)	245 (97.2)	72 (89.9)	0.033
Anticoagulant (%)	107 (1.5)	34 (13.5)	26 (32.5)	<0.001
Warfarin	100 (1.4)	33 (13.1)	26 (32.5)	<0.001
NOAC	7 (0.1)	1 (0.4)	0 (0.0)	0.310
DAPT plus anticoagulant (%)	97 (1.4)	34 (13.5)	23 (28.8)	<0.001
Statin (%)	6747 (96.5)	246 (97.7)	77 (96.1)	0.632
Beta-blocker (%)	6135 (87.7)	209 (82.8)	67 (84.2)	0.060
ACE inhibitor or ARB (%)	6425 (91.9)	237 (94.1)	71 (89.1)	0.346

* $p < 0.05$ between Non-AF vs. NOAF; † $p < 0.05$ between Non-AF vs. Previous AF ‡ $p < 0.05$ between NOAF vs. Previous AF.

Table S4. Characteristics according to onset time of AF in women (n = 2810).

Variables	Non-AF (n = 2,642)	NOAF (n = 118)	Previous AF (n = 50)	p Value
Age (years)	70.8 ± 10.5	74.1 ± 9.1 *	75.5 ± 6.5 †	<0.001
Body mass index (kg/m²)	23.6 ± 3.6	23.4 ± 3.7	24.5 ± 5.2	0.192
Diabetes mellitus (%)	1,026 (38.8)	39 (33.1)	24 (48.0)	0.180
Hypertension (%)	1757 (66.5)	89 (75.4)	37 (74.0)	0.075
Dyslipidemia (%)	454 (17.2)	20 (16.9)	5 (10.0)	0.408
Chronic kidney disease (%)	59 (2.2)	2 (1.7)	5 (10.0)	0.010
Current smoker (%)	260 (9.8)	11 (9.3)	1 (2.0)	0.164
Family History of CAD (%)	48 (1.8)	0 (0.0)	1 (2.0)	0.335
Previous HF (%)	53 (2.0)	5 (4.2)	7 (14.0)	<0.001
Previous MI (%)	88 (3.3)	7 (5.9)	2 (4.0)	0.224
Previous PAD (%)	10 (0.4)	2 (1.7)	0 (0.0)	0.158
Previous stroke (%)	222 (8.4)	14 (11.9)	7 (14.0)	0.129
SBP at admission (mmHg)	129 ± 27	119 ± 27 *	136 ± 29 ‡	<0.001
DBP at admission (mmHg)	77 ± 16	74 ± 18 *	83 ± 17	0.003
HR at admission (bpm)	80 ± 19	85 ± 26 *	88 ± 27 †	<0.001
Killip class ≥ 2 (%)	742 (28.1)	60 (51.4)	18 (36.4)	<0.001
LV ejection fraction (%)	52.7 ± 11.4	51.6 ± 12.7	50.0 ± 12.4	0.165
LA AP diameter (mm)	36.6 ± 6.1	39.7 ± 8.4*	47.3 ± 7.6 †,‡	<0.001
$eGFR_{MDRD}$ (mL/m²/1.73m²)	69.7 ± 27.7	67.0 ± 25.2	52.9 ± 25.9 †,‡	<0.001
HbA1c (%)	6.8 ± 1.5	6.7 ± 1.4	6.9 ± 1.2	0.781
Total cholesterol (mg/dL)	182.2 ± 46.8	170.0 ± 46.8 *	163.4 ± 48.1†	0.001
Triglyceride (mg/dL)	116.0 ± 71.0	95.0 ± 51.7*	104.4 ± 63.5	0.005
HDL cholesterol (mg/dL)	43.1 ± 11.9	42.0 ± 11.5	42.7 ± 10.1	0.610
LDL cholesterol (mg/dL)	115.8 ± 40.5	108.3 ± 43.0	103.9 ± 40.3	0.030
Peak CK (U/L)	327 (121,1032)	400 (119,1283)	188 (83,397)‡	0.038
Peak CK-MB (μg/mL)	34.7 (11.4,115.6)	36.3 (12.0,106.4)	10.8 (5.1,41.1) ‡	0.025
Peak TnI (ng/mL)	14.6 (3.5,50.0)	17.2 (4.4,41.3)	7.0 (1.7,26.7)	0.249
STEMI (%)	1267 (48.0)	52 (44.1)	20 (40.0)	0.391
Extent of CAD (%)				
One vessel disease	1100 (41.6)	55 (46.6)	13 (26.0)	0.044
Two vessel disease	863 (32.7)	43 (36.4)	22 (44.0)	0.174
Three vessel disease	679 (25.7)	20 (16.9)	15 (30.0)	0.077
CABG (%)	6 (0.2)	0 (0.0)	0 (0.0)	1.000
CHA_2DS_2-VASc score	4.2 ± 1.8	4.9 ± 1.9*	5.1 ± 1.9†	<0.001
Medications at discharge (%)				
Aspirin (%)	2,596 (98.3)	113 (95.8)	46 (92.0)	0.004
P_2Y_{12} inhibitor (%)	2,522 (95.5)	116 (98.3)	48 (96.0)	0.346
DAPT (%)	2,496 (94.5)	111 (94.1)	46 (91.8)	0.554
Anticoagulant (%)	49 (1.9)	27 (22.9)	14 (28.0)	<0.001
Warfarin	44 (1.7)	27 (22.9)	13 (26.0)	<0.001
NOAC	5 (0.2)	0 (0.0)	1 (2.0)	0.124
DAPT plus anticoagulant (%)	44 (1.7)	24 (20.3)	14 (28.0)	<0.001
Statin (%)	2509 (95.0)	117 (99.0)	45 (91.7)	0.052
Beta-blocker (%)	2264 (85.7)	104 (87.9)	40 (80.4)	0.483
ACE inhibitor or ARB (%)	2373 (89.8)	113 (95.9)	46 (92.7)	0.109

* $p < 0.05$ between Non-AF vs. NOAF; † $p < 0.05$ between Non-AF vs. Previous AF; ‡ $p < 0.05$ between NOAF vs. Previous AF.
